# 

**Published:** 1878-10

**Authors:** 


### CONTENTS:

Article I.—American Dental Association (Continued.) .......... 241

Article II.—Relations of the Dental Pulp to the other Tooth Tissues (Continued.) By L. C. Ingersoll .......... 256

Article III.—Gold can be so Manipulated as to make Operations Permanent. By M. H. Webb, D. D. S. .......... 268

Article IV.—Substance of Lectures on Gold (Continued.) By Prof. Hodgkin .......... 272

Article V.—Virginia State Dental Association .......... 275

Article VI.—Results of Some Recent Experiments .......... 277

Article VII.—Georgia State Dental Society .......... 279

### EDITORIAL, ETC.

Qualification of the Dental Student .......... 280

The New Rubber Suit .......... 282

### MONTHLY SUMMARY.

Nature and Treatment of Neuralgia .......... 283
Chloral Hydrate in Tetanus .......... 283
Treatment of Wounds .......... 284
Hypodermic Injections of Chloroform .......... 284
Sulphate of Quinine .......... 284
A Rival to Carbolic Acid .......... 285
Salicylic Acid and Borax .......... 285
Abscess of Lower Lip from Paris Green .......... 285
Antiseptic Solution of Benzoic Acid .......... 286
Combined Use of Chloroform and Morphia .......... 286
Prevention of Chloroform Narcosis .......... 286
Abortive Treatment of Furunculus .......... 287
A New Cheap and Self-Generating Disinfectant .......... 287
Howard's Method of Artificial Respiration .......... 288

